# Innate and adaptive immune dysregulation in critically ill ICU patients

**DOI:** 10.1038/s41598-018-28409-7

**Published:** 2018-07-05

**Authors:** Niharika Arora Duggal, Catherine Snelson, Ulfath Shaheen, Victoria Pearce, Janet M. Lord

**Affiliations:** 1 Institute of Inflammation and Ageing, University of Birmingham, Birmingham, UK; 2 NIHR Surgical Reconstruction and Microbiology Research Centre, University Hospital Birmingham, Mindelsohn Way, Birmingham, UK; 3 NIHR Birmingham Biomedical Research Centre, University Hospital Birmingham, Mindelsohn Way, Birmingham, UK

## Abstract

This study aimed to evaluate whether ICU patients who developed persistent critical illness displayed an immune profile similar to an aged immune phenotype and any associations with patient outcomes. Twenty two critically ill ICU patients (27–76 years, 15 males), at day 5 of mechanical ventilation, and 22 healthy age-matched controls (27–77 years, 13 males) were recruited. Frequency and phenotype of innate and adaptive immune cells and telomere length in peripheral blood mononuclear cells (PBMCs) were measured. An elevated granulocyte count (p < 0.0001), increased numbers of immature granulocytes (p < 0.0001), increased CD16^++ve^ monocytes (p = 0.003) and CD14^+ve^ HLADR^dim/low^ monocytes (p = 0.004) and lower NK cell numbers (p = 0.007) were observed in ICU patients compared to controls. Critically ill patients also had lower numbers of total T lymphocytes (p = 0.03), naïve CD4 T cells (p = 0.003) and PTK7^+ve^ recent thymic emigrants (p = 0.002), and increased senescent CD28^−ve^ CD57^+ve^ CD4 T cells (p = 0.02), but there was no difference in PBMC telomere length. Regulatory immune cell frequency was affected with reduced circulating CD19^+ve^CD24^hi^CD38^hi^ regulatory B cells (p = 0.02). However, only a raised neutrophil:lymphocyte ratio and reduced frequency of CD14^+ve^ HLADR^dim/low^ monocytes were associated with poor outcomes. We conclude that persistent critical illness results in changes to immune cell phenotype only some of which are similar to that seen in physiological ageing of the immune system.

## Introduction

According to the Hospital Episode Statistics Analysis report for 2014, approximately 250,000 patients were admitted to intensive care units (ICU) in the UK^1^. Patients in these wards are heterogeneous clinically and suffer from life threatening conditions including: sepsis/infection; renal failure; cardiac surgery and major trauma.

Critically ill patients often show symptoms typically involving an initial systemic inflammatory response syndrome (SIRS), characterised by the release of pro-inflammatory mediators^2^. SIRS is initially caused by non-infective events, such as cardiogenic shock, resuscitation, surgery, or trauma-related tissue damage and affects almost half of patients admitted to ICU^3^. SIRS is accompanied by a compensatory anti-inflammatory response syndrome (CARS) initiated to dampen the inflammatory process and aid return to homeostasis^4^. Either an excessive CARs or an insufficient SIRs response will render the host susceptible to infections or unable to clear existing infections^5^. Nosocomial infections in critically ill patients are associated with an increased length of hospital stay, elevated health care costs and increased mortality^6^.

Persistent critical illness can be defined as occurring when a patient’s reason for being in ICU is more related to their ongoing critical illness than their original reason for admission^7^. This has been shown to occur by day 10 of ICU, at which time antecedent patient characteristics such as age, sex and chronic health status predict survival more accurately than reason for admission and physiological derangement^8^. These patients have a higher mortality and consume significant resource, so a better understanding of the pathophysiology of persistent critical illness is required.

Immunoparesis is seen post critical illness and involves alterations in both innate and adaptive immune responses, including neutrophil dysfunction^9^, altered monocyte phenotype and antigen presentation capacity^10^, lymphopenia and impaired lymphocyte responses to novel challenge^11^ and elevated pro-inflammatory cytokines^12^. The clinical consequences of immune suppression in the ICU setting include increased risk of multiple organ failure, infections and mortality^13–15^. A balanced systemic host immune response is necessary to cope with critical illness and improved understanding of the effect of persistent critical illness on immunity will potentially identify novel prognostic biomarkers and routes to therapy.

Although there are some studies that have looked at immune compromise following major trauma, including from our own group^16,17^, there are no comprehensive studies exploring the effect of persistent critical illness in its broadest context on innate and adaptive immune cells. In this study we aimed to carry out a detailed assessment of the composition of the innate (monocyte, NK cells) and adaptive (T cells and B cells) arms of the immune system in a critical care population. We specifically chose a population most at risk of persistent critical illness and the consequent high mortality and long term sequelae by recruiting patients who had been mechanically ventilated for 5 days.

Lastly, physiological ageing is accompanied by significant remodelling of the immune system termed immunesenescence, which includes: thymic atrophy leading to a reduced output of naïve T cells; increased frequency of senescent T cells with shortened telomeres; reduced regulatory cell function; skewing of haemopoiesis towards myeloid cell generation; reduced innate cell bactericidal function, and increased systemic inflammation^18^. The clinical consequences of immunesenescence include increased risk of infections, autoimmune disease and chronic inflammatory disease^19^, which might also be a contributing factor towards the poor outcome observed in ICU patients. We thus also attempted to test the hypothesis that persistent critically ill ICU patients develop an aged immune phenotype and identify potential immune biomarkers that could predict outcome in critically ill patients.

## Results

### Participant demographics

The baseline characteristics of 22 critically ill patients (range 27–76 years, 15 males) and 22 age matched healthy controls (range 27–77 years, 13 males) are shown in Table 1. On admission to the ICU patients had a mean APACHE II score of 18.13 ± 6.62 and on day 5 they had a mean SOFA score of 10.73 ± 4.93. Patients had an average length of stay in ICU of 20.94 days. Sepsis was the major primary diagnosis (8 patients).

**Table 1:** Patient demographics.

	ICU patients (n = 22)	Healthy controls (n = 22)
Age	59.32 ± 13.82	61.23 ± 0.14.88
Male gender, n (%)	15 (68.18%)	13 (59%)
Mortality, n (%)	2 (9.09%)	x
SOFA score	10.73 ± 4.93	x
APACHE II score	18.13 ± 6.62	x
ICU Length of stay (days)	20.94 ± 12.71	x
Primary diagnosis		
Sepsis	8 (36.3%)	
Cardiac surgery	3 (13.6%)	
Poly trauma	3 (13.6%)	
Kidney failure	3 (13.6%)	
Respiratory failure	1 (4.54%)	
Liver Cirrhosis	1 (4.54%)	
Other	3 (13.6%)	

### Neutrophil count and neutrophil:lymphocyte ratio

**Figure 1:**
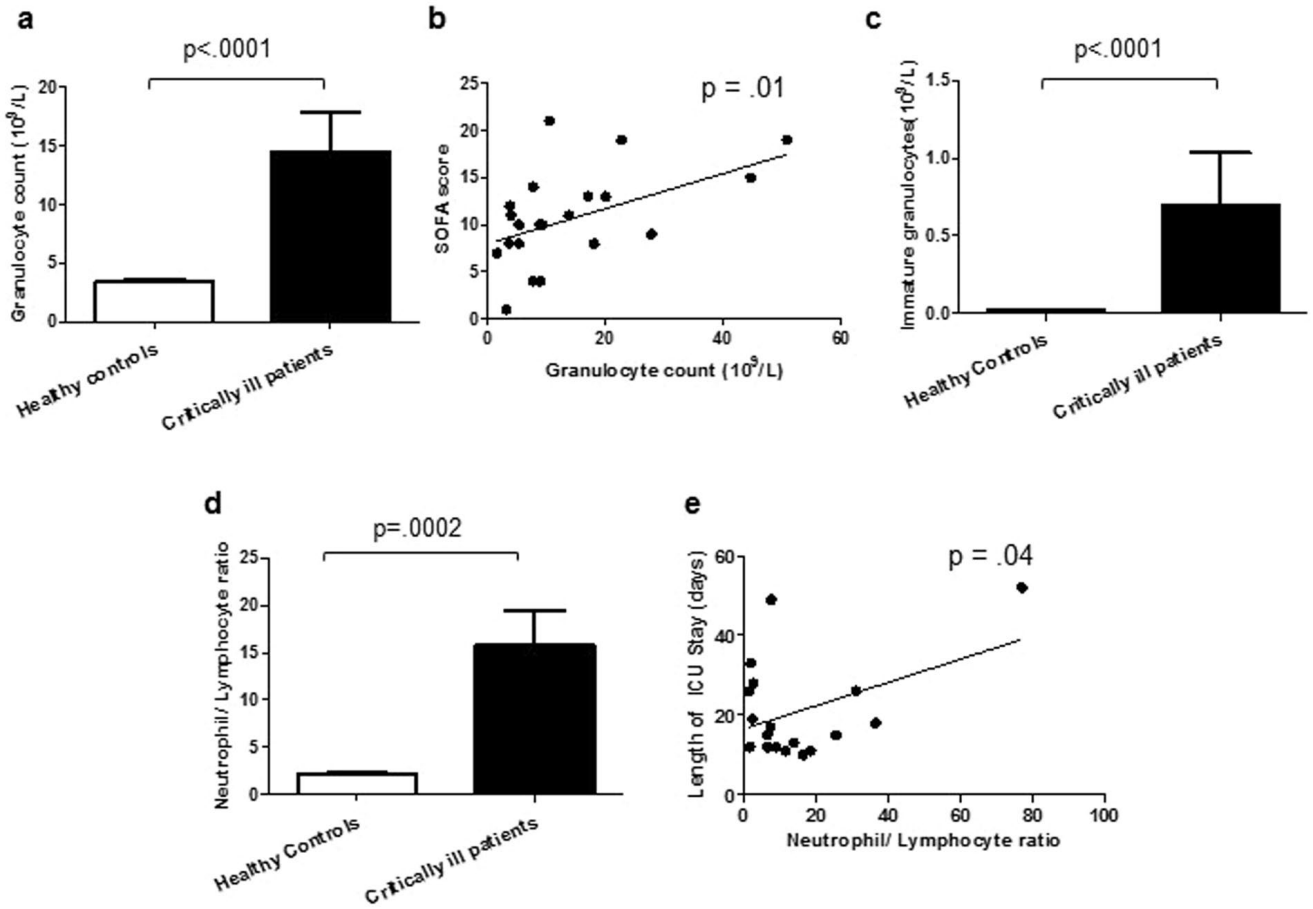
Neutrophil count and neutrophil:lymphocyte ratio. (**a**) Blood neutrophil count in critically ill patients (n = 22) and healthy controls (n = 22). (**b**) Correlation between neutrophil count and SOFA score in critically ill patients (n = 20). (**c**) Neutrophil:lymphocyte ratio in critically ill patients (n = 22) and healthy controls (n = 22). (**d**) Correlation between neutrophil:lymphocyte ratio and ICU length of stay (days) in critically ill patients (n = 19). Data are expressed as mean ± SEM.

The peripheral granulocyte count was significantly higher in critically ill patients compared to healthy controls, p < 0.0001 (Fig. 1a) and there was a significant positive correlation between granulocyte numbers and SOFA score, p = 0.01 R^2^ = 0.25 (Fig. 1b). Additionally, the number of immature granulocytes, which have reduced bactericidal function, in critically ill patients was significantly higher than in healthy controls, p < 0.0001 (Fig. 1c). The neutrophil:lymphocyte ratio was significantly higher in critically ill patients compared to controls, p = 0.0002 (Fig. 1d) and was positively associated with ICU length of stay, p = 0.04 R^2^ = 0.19 (Fig. 1e).

### Monocyte subset distribution in critically ill patients

**Figure 2:**
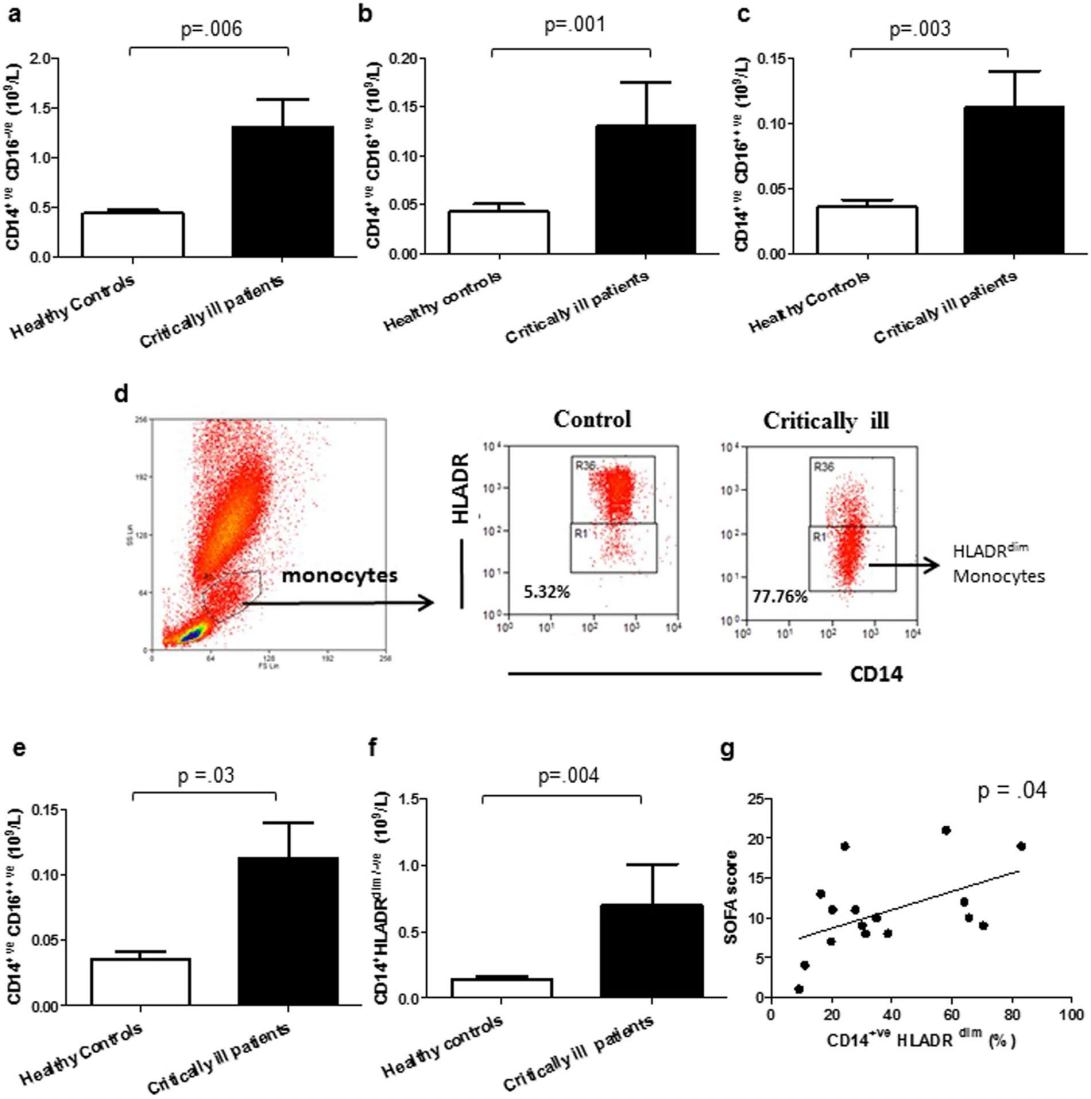
Monocyte subset distribution in critically ill patients (**a**) Blood CD14^+ve^ CD16^−ve^; (**b**) CD14^+ve^ CD16^+ve^; (**c**) CD14^+ve^ CD16^++ve^ monocyte counts in critically ill patients at recruitment (n = 20) and healthy controls (n = 19). (**d**) Whole blood was stained with anti-CD14 and HLADR antibodies and monocytes (CD14^+^) gated to identify HLADR^dim^ monocytes. (**e**) Blood frequency and (**f**) numbers of CD14^+ve^ HLADR^dim^ monocytes in critically ill patients (n = 20) and healthy controls (n = 19). The mean value is indicated by the bar. (**g**) Correlation between frequency of CD14^+ve^ HLADR^dim^ monocyte and SOFA score in critically ill patients (n = 17). Data are expressed as mean ± SEM.

The total monocyte count was also significantly higher in critically ill patients compared to age matched healthy controls, p = 0.008. The differential expression of CD14 and CD16 has been used to define three peripheral subsets of monocytes: classical (CD14^+^ CD16^−^) represent approximately 90% of monocytes in a healthy individual; intermediate (CD14^+^ CD16^+^) and non-classical monocytes (CD14^+^ CD16^++^) make up the remaining fraction^20^. On comparing the peripheral frequency of CD16^−ve^, CD16^+ve^ and CD16^++ve^ monocytes between controls and critically ill patients no significant difference was observed, p = 0.48, p = 0.67 and p = 0.15 respectively. However, the absolute numbers of CD16^−ve^, CD16^+ve^ and CD16^++ve^ monocytes were higher in critically ill patients, p = 0.006, p = 0.001 and p = 0.003 respectively (Fig. 2a–c).

CD14^+ve^ HLADR^dim/low^ monocytes have been reported to have an immunosuppressive phenotype^21^. The gating used to identify these cells is shown in Fig. 2d. The frequency (Fig. 2e) and absolute numbers (Fig. 2f) of CD14^+ve^ HLADR^dim/low^ monocytes was higher in critically ill patients compared to controls, p = 0.03, p = 0.004 respectively. Interestingly, a positive correlation was observed between the frequency of CD14^+ve^ HLADR^dim/low^ monocytes and SOFA score in ICU patients, p = 0.04 R^2^ = 0.25 (Fig. 2g).

### NK cells in critically ill patients

**Figure 3:**
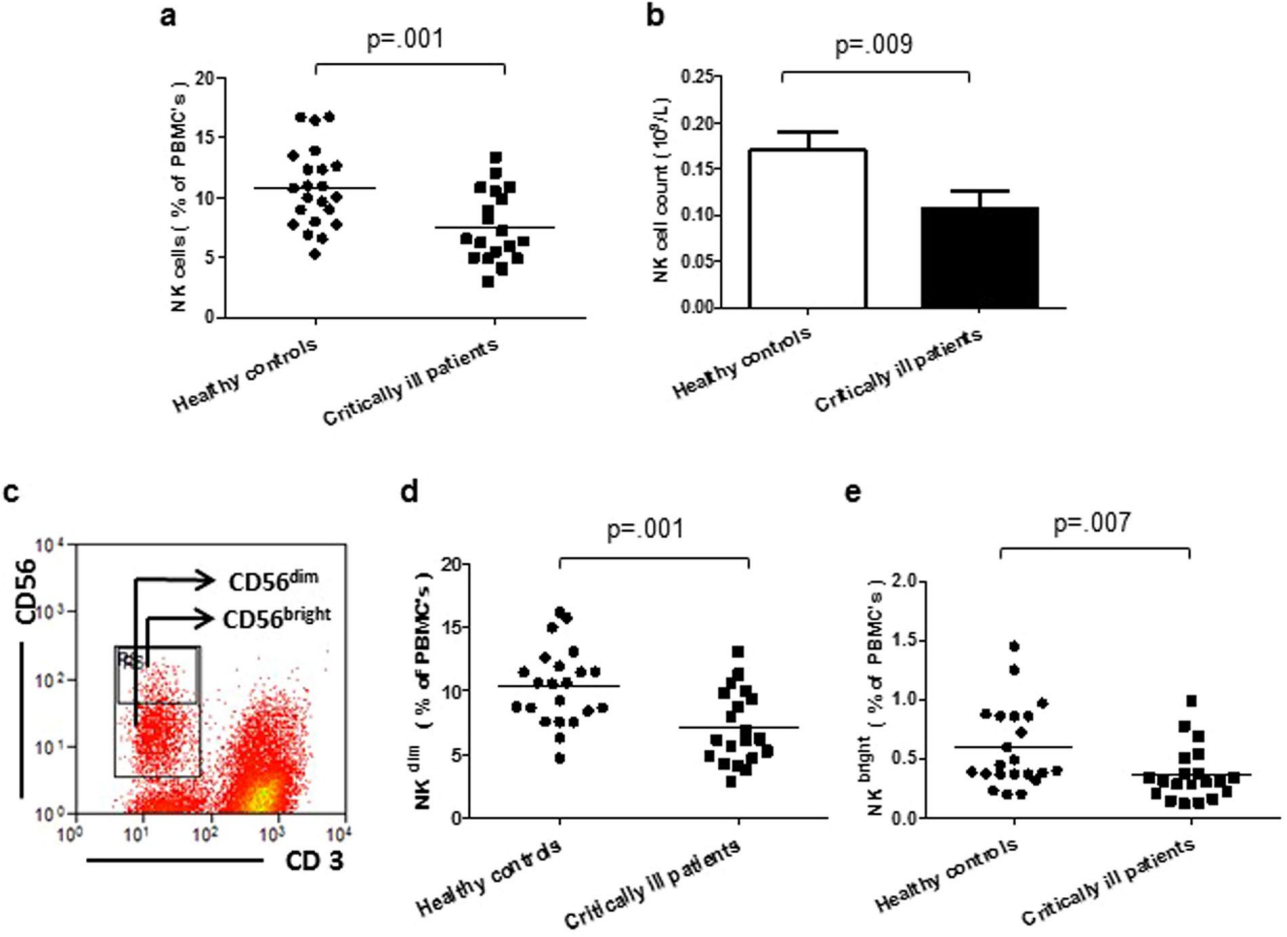
NK cell subsets in critically ill patients (**a**) Blood frequency and (**b**) numbers of NK cells in critically ill patients (n = 22) and healthy controls (n = 22). Data are expressed as mean ± SEM. (**c**) PBMCs were stained with anti-CD3 and anti-CD56 antibodies to identify CD56^dim^ and CD56^bright^ NK cells. (**d**) Frequency of CD56^dim^ NK cells and (**e**) CD56^bright^ NK cells in critically ill patients (n = 22) and healthy controls (n = 22).

A significant reduction in the frequency of NK cells in the PBMC pool, p = 0.001 (Fig. 3a) and the absolute numbers of these cells p = 0.009, (Fig. 3b) was observed in critically ill patients. NK cells have been divided into two subsets on the basis of their relative expression of CD56, namely CD56^dim^ and CD56^bright^. The gating strategy used to identify these subtypes is shown in Fig. 3c. 85% of NK cells are CD56^dim^ and these cells have high cytotoxic potential, their frequency p = 0.001 (Fig. 3d) and absolute numbers p = 0.002 were significantly reduced in critically ill patients. Similarly, a lower frequency p = 0.007 (Fig. 3e) and absolute numbers of CD56^bright^ NK cells p = 0.008 was also seen in critically ill patients.

### T cell subset distribution in critically ill patients

**Figure 4:**
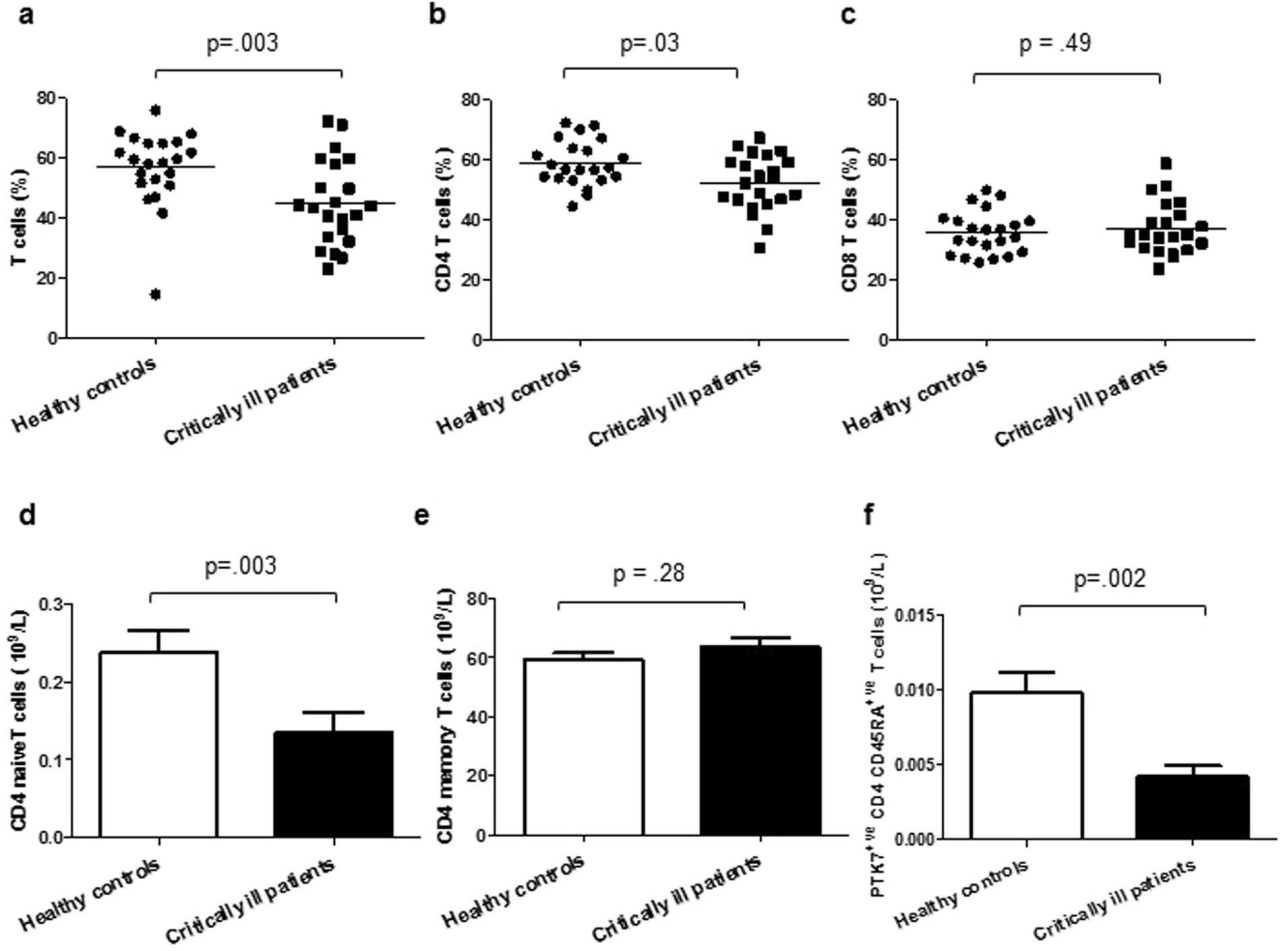
T cell subset distribution in critically ill patients (**a**) Frequency of total CD3^+ve^ T cells, (**b**) CD4 T cells, (**c**) CD8 T cells in critically ill patients (n = 22) and healthy controls (n = 22). Numbers of (**d**) Naïve CD4 T cells, (**e**) memory CD4 T cells, (**f**) PTK7^+ve^ CD45RA CD4 T cells in critically ill patients (n = 22) and healthy controls (n = 22). Data are expressed as mean ± SEM.

Functional subsets of T cells include CD4^+ve^ T helper cells and CD8^+ve^ cytotoxic cells. The frequency p = 0.003 (Fig. 4a) and absolute number of T cells, p = 0.03 was lower in critically ill patients. This was driven by a significant reduction in the frequency p = 0.03 (Fig. 4b) and absolute numbers, p = 0.004 of CD4 T cells in critically ill patients. No significant differences were observed for CD8 T cells between critically ill patients and healthy controls (Fig. 4c).

We examined the CD4 T cell population further and found that the frequency of naïve CD4 T cells was not significantly different in critically ill patients, p = 0.42, but the absolute cell count was lower compared to healthy controls, p = 0.003 (Fig. 4d). No significant differences were observed in frequency, p = 0.19 or absolute numbers of CD4 memory T cells, p = 0.28 (Fig. 4e). Overall, the CD4 naïve:memory ratio did not differ between healthy controls and critically ill patients, p = 0.36. PTK7 has been identified as a novel marker for the identification of CD4 recent thymic emigrants (RTEs) indirectly indicating thymic output^22^. To determine the contribution of RTEs and reduced thymic output to the fall in naïve CD4 T cells PTK7^+ve^ cells were enumerated and found to be lower in critically ill patients, p = 0.002 (Fig. 4f).

### Senescent T cells

**Figure 5:**
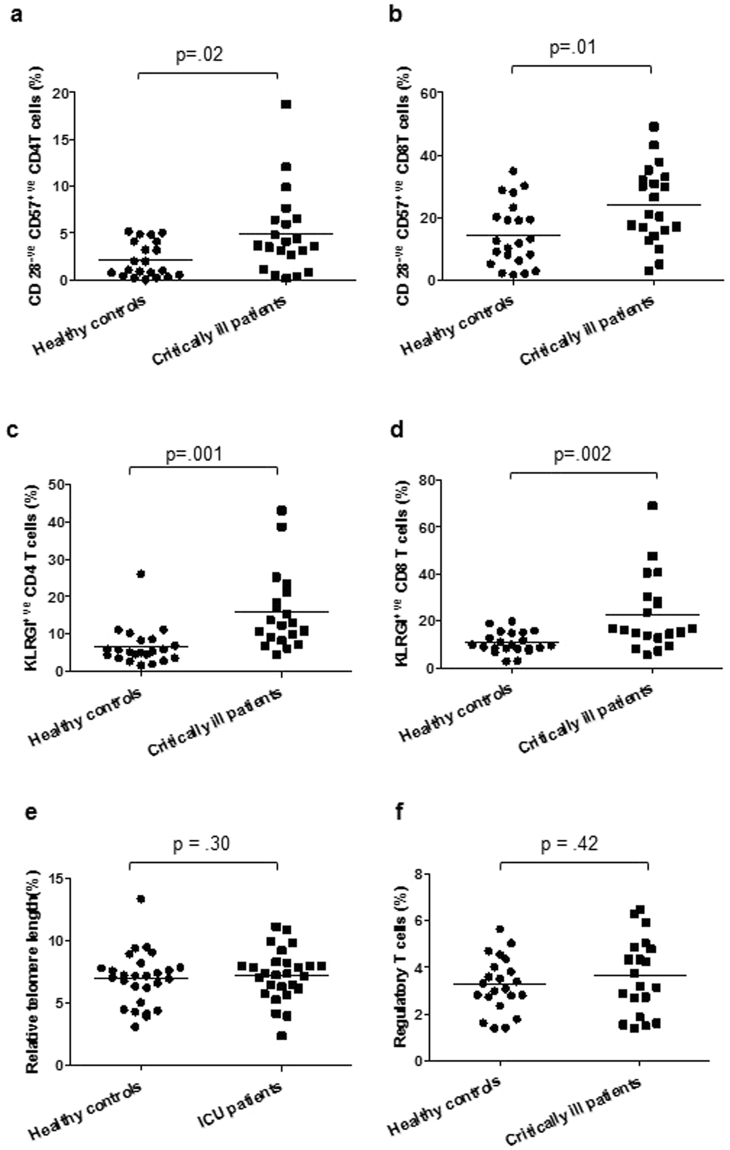
Accumulation of senescent T cells in critically ill patients (**a**) Frequency of CD28^−ve^ CD57^+ve^ CD4 T cells, (**b**) CD28^−ve^ CD57^+ve^ CD8 T cells, (**c**) KLRG1^+ve^ CD4 T cells, (**d**) KLRG1^+ve^ CD8 T cells in critically ill patients (n = 22) and healthy controls (n = 22). (**e**) Relative telomere length (RTL) in PBMCs for ICU patients (n = 22) compared to age-matched healthy controls (n = 22). (**f**) Frequency of CD25^+ve^ Foxp3^+ve^ CD4 Regulatory T cells in critically ill patients (n = 22) and healthy controls (n = 22). The mean value is indicated by the bar.

T cells have a finite replicative potential, and repeated cell division results in telomere shortening and functional senescence. The loss of CD28, gain of CD57 and killer cell lectin–like receptor (KLRG1) are cell surface markers used to identify senescent T cells in humans^23^. The frequency of CD28^−ve^ CD57^+ve^ CD4 T cells, p = 0.02 (Fig. 5a) and CD28^−ve^ CD57^+ve^ CD8 T cells, p = 0.01 (Fig. 5b) was significantly higher in critically ill patients. However, the absolute numbers of CD28^−ve^ CD57^+ve^ CD4 T cells, p = 0.52 and CD28^−ve^ CD57^+ve^ CD8 T cells did not differ in critically ill patients, p = 0.59. Similarly, the frequency of KLRG1^+ve^ CD4 T cells and KLRG1^+ve^ CD8 T cells was higher in critically ill patients compared to healthy controls, p < 0.001 (Fig. 5c) and p = 0.002 (Fig. 5d) respectively. Again, the absolute numbers of KLRG1^+ve^ CD4 T cells, p = 0.41 and KLRG1^+ve^ CD8 T cells, p = 0.60 were not different in the critically ill patients.

### Telomere length

Relative telomere length (RTL) in PBMCs compared to the 1301 control cell line was calculated for critically ill patients and healthy controls but no significant differences were observed, p = 0.26 (Fig. 5e).

### Regulatory T cells

T_regs_ have been identified as a subpopulation of T cells expressing CD4, CD25 and the transcription factor Foxp3 that plays an essential role in T_reg_ cell differentiation and development^24^. On examining the frequency of T_regs,_ no differences were observed between healthy controls and critically ill patients, p = 0.57 [Fig. 5f].

### B cell subset distribution in critically ill patients

**Figure 6:**
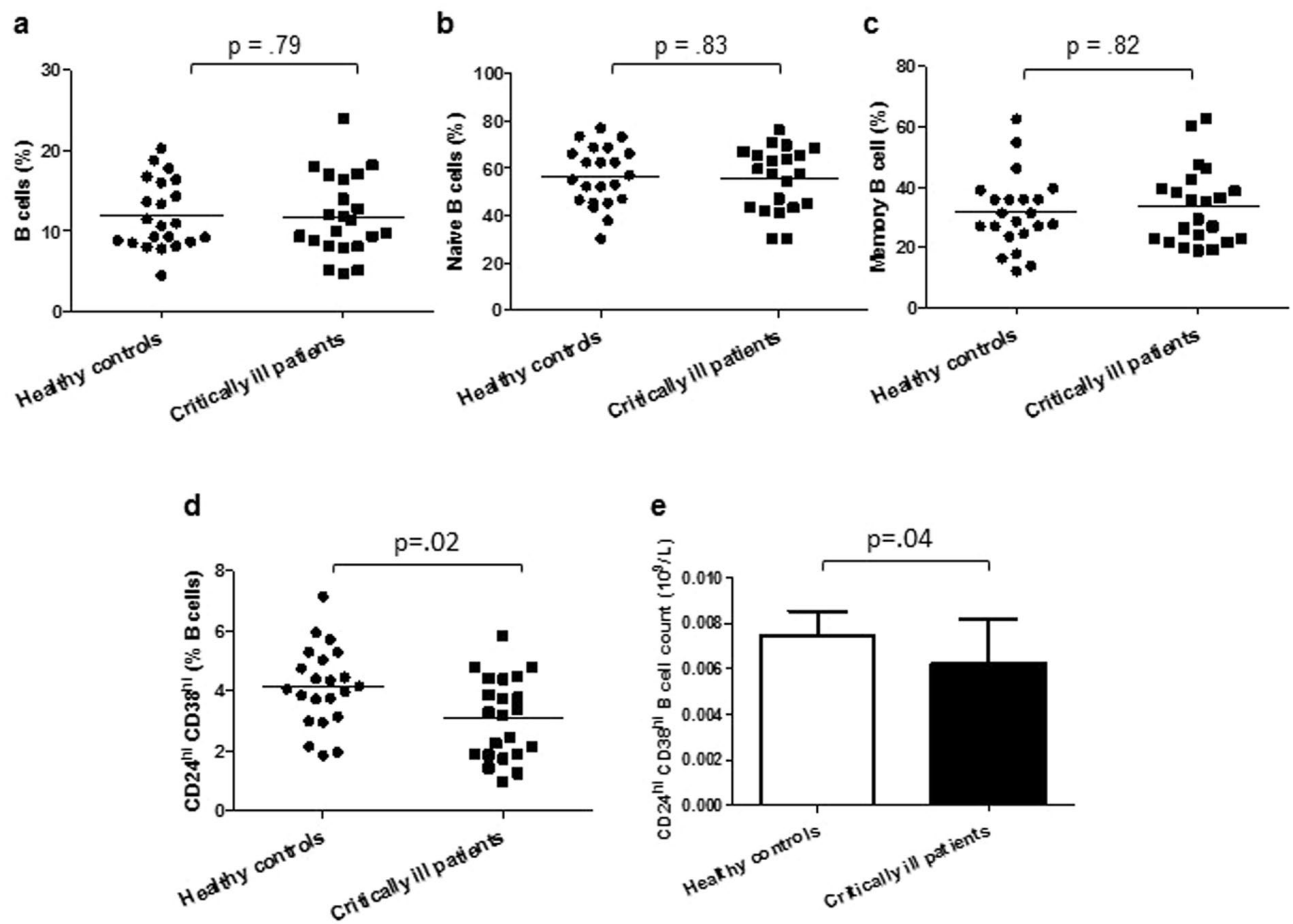
B cell subset distribution in critically ill patients Frequency of (**a**) CD19^+ve^ B cells, (**b**) naïve IgD^+ve^ CD27^−ve^ CD19^+ve^ B cells, (**c**) total memory B cells, (**d**) CD19^+ve^CD24^hi^CD38^hi^ B cells in critically ill patients (n = 22) and healthy controls (n = 22). The mean value is indicated by the bar. The absolute number of (**e**) CD19^+ve^CD24^hi^CD38^hi^ B cells in peripheral blood of healthy young (n = 22) and old donors (n = 22). Data are expressed as mean ± SEM.

B cells can be divided into three subsets on the basis of CD27 and IgD expression; naïve (CD27^−ve^ IgD^+ve^), switched memory (CD27^+ve^ IgD^−ve^) and unswitched memory (CD27^+ve^ IgD^+ve^) B cells^25^. Total B cell frequency, p = 0.84 (Fig. 6a) and absolute numbers did not differ between our two groups, p = 0.49. The frequency of naïve B cells, p = 0.73 (Fig. 6b) and total memory B cells (switched and unswitched memory B cells), p = 0.79 (Fig. 6c) also showed no significant differences between the groups. Transitional B cells (CD19^+ve^CD24^hi^CD38^hi^) have been reported to exert immunosuppressive properties mainly via IL10 production^26^. CD24^hi^CD38^hi^ B cell frequency, p = 0.02 (Fig. 6d) and absolute numbers, p = 0.04 (Fig. 6e) in peripheral blood were significantly lower in critically ill patients.

## Discussion

Immunological dysregulation in critically ill patients is a serious challenge in critical care management because its occurrence increases risk of secondary infections in ICU patients, which increases their length of stay and health care costs. Patients with persistent critical illness have a high mortality and a high risk of long term morbidity. From this perspective developing an understanding of the underlying immune response in persistent critical illness in its broadest context is essential to improving the management of these patients and avoiding complications such as sepsis.

One of the hypotheses we were testing here was that the immune system of the persistent critically ill patient may develop an aged phenotype. It is well established that the immune system declines with age, termed immunesenescence^18^ and that this contributes to increased risk of infection. Key features of immunesenescence are reduced innate cell function^27,28^, a reduced naïve:memory T cell ratio and thymic output, shorter lymphocyte telomeres, increased numbers of functionally senescent T cells^29^ and reduced B regulatory lymphocyte frequency and function^30^. In this study of 22 ICU patients we found some characteristics of the aged immune phenotype, most notably in the lymphocyte compartment, but not all suggesting that overt acceleration of immune ageing in persistent critical illness does not occur and may be restricted to specific features of adaptive immunity.

We observed a decline in peripheral T cell numbers, which has also been reported during sepsis and SIRS^31^. Here lymphopenia was associated with increased ICU length of stay and in trauma patients it has been reported previously to be associated with mortality and multiple organ dysfunction^32^. Interestingly we found few changes in T cell subsets, with the exception of naïve T cells whose frequency declined in ICU patients. Such cells are crucial for responding to new antigens and vaccinations and their numbers decline with age due to atrophy of the thymus^33^, the organ in which T cells develop. To determine if this fall in naïve T cells could be due to the impact of critical illness on thymic function, a potentially irreversible effect, we measured levels of recent thymic emigrants in our patients and found them to be lower than in healthy controls. Our data are in line with findings in murine models of sepsis and traumatic injury which reported increased thymic atrophy and reduced thymic output of naïve T cells to the periphery^34,35^. Lastly, an accumulation of senescent (CD28^−ve^CD57^+ve^KLRG1^+ve^) T cells also occurred during critical illness, which has been previously reported in sepsis patients^36^. The T cell compartment thus showed the clearest hallmarks of an aged phenotype.

Regulatory T cells^24^ and B cells^26^ play a key role in immune homeostasis. Higher circulating T_reg_ levels have been observed in trauma patients^37^, but we did not see this in our cohort of critically ill patients. The role of B_regs_ in orchestrating critical illness induced immunoparesis has not been explored and here we report a decline in circulating B_regs_ in critically ill patients. Although we did not examine IL10 production by these B_regs_, there has been a recent study reporting a decline in IL10 producing B cells in brain injury patients^38^. As the frequency and function of these cells declines with age^30^, this may represent further evidence of immune ageing in the lymphocyte compartment.

Neutrophils are key contributors to the immediate innate immune response and we confirmed the well documented neutrophilia in our critically ill patients. Interestingly we also found an increase in immature granulocytes in blood, which has been reported in major trauma patients^39^ but not previously in the broad setting of ICU patients. Immature granulocytes are less efficient than mature neutrophils in their bactericidal function^40^. An increase in these cells may result in delayed bacterial clearance and increase the risk of infections in ICU patients, though a much larger study would be needed to test this proposal. We also identified a raised neutrophil:lymphocyte ratio and this was associated with increased length of ICU stay. Others have found this ratio to be a prognostic indicator of mortality in critically ill patients^41^ and patients with sepsis^42^. In a study of 130 septic shock patients a low neutrophil:lymphocyte ratio upon admission was associated with early death but late death was associated with a raised ratio in the first 5 days after admission^42^. Interestingly a raised neutrophil:lymphocyte ratio is a feature of immunesenescence due to altered haemopoiesis favouring the myeloid lineage^43^. In ICU patients this is most likely due to the transient neutrophilia induced by surgery, sepsis or chronic disease in these patients.

In relation to monocytes we found an expansion of the inflammatory CD16^+ve^ monocyte subset, which has also been observed in sepsis patients^44^ and in ageing^45^. Monocytes are known to activate the adaptive immune response via presentation of antigens on cell surface class II MHC molecules (HLADR). We found an elevated frequency of an immunosuppressive HLADR^dim/−ve^ monocyte subset in critically ill patients. Reduced monocyte HLADR expression has been previously reported in trauma^46^ and sepsis patients^47,48^ and has also been proposed as a marker of immune deficiency. Interestingly, we have also observed a positive association between SOFA score and the frequency of HLADR^dim/−ve^ monocytes which has also been previously reported in sepsis patients^49^.

Natural Killer (NK) cells are cytotoxic cells that are responsible for killing virus infected cells and are also an important source of inflammatory cytokines, such as IFNγ^50^. We report here a state of NK cell lymphopenia in critically ill patients, which has been previously reported in sepsis patients^51^ and also recently in traumatic injury patients^16^ and might be due to rapid recruitment of NK cells to sites of inflammation. We did not however find an association between NK cell numbers and patient outcomes.

This study has a number of limitations. Firstly, only a small number of ICU patients, 22, were enrolled in this study and it was a single centre study. However the heterogeneity of ICU admission causes, ranging from cardiac arrest to liver cirrhosis suggests the broad generalisability of the data. Secondly, immune parameters were only measured at day 5 post ventilation and we were not able to follow ICU patients after hospital discharge to determine whether immune suppression recovered long term post ICU discharge. There were also issues with obtaining sufficient blood for all of the assays that we had planned and thus there are uneven numbers for some of the measures examined. Finally, we did not measure immune cell function, only phenotype and future studies should carry out such measures to confirm reduced immunity in these patients.

We conclude that persistent critical illness induces some remodelling of the adaptive immune cell populations, with evidence of a phenotype similar to age-related immune decline in lymphocytes, notably reduced thymic output, increased frequency of senescent T cells and reduced regulatory B cells. We have identified an increased neutrophil:lymphocyte ratio and loss of monocyte HLADR expression as biomarkers for increased length of stay in critically ill patients.

## Methods

### Participants and study design

This was a single centre observational study in critically ill patients (n = 22) admitted to the ICU wards of the Queen Elizabeth Hospital, Birmingham between January and September 2016 that were a part of a trial of rehabilitation in the ICU (ISRCTN90103222). Ethical approval was granted by the East Midlands Nottingham and the University of Birmingham Research Ethics Committees. Recruitment of ICU patients was conducted on day 5 of mechanical ventilation of the patients. All patients lacked capacity at the time of recruitment. Written informed assent was gained from the patient’s advocate or if no advocate then an independent Registered Medical Practitioner gave their assent. If the patient later regained capacity then written consent was sought for agreement to continue in the study. 22 healthy age-matched controls were also recruited and gave written informed consent for their participation in the study. The baseline demographics, cause of admission and clinical history of all patients were recorded. Disease severity was quantified upon admission to ICU using the Acute Physiology and Chronic Health Evaluation II (APACHE II)^52^ and then reassessed on day 5 using the Sequential Organ Failure Assessment score (SOFA)^53^. Blood samples were taken on recruitment to the study. All experiments were performed in accordance with the study protocol and all relevant guidelines and regulations.

### Blood sampling and monocyte phenotyping

Venous blood samples (6 ml) were collected in lithium heparin coated vacutainers. Whole blood counts were performed using a Sysmex XN-1000 haematology analyser (Sysmex, UK). The blood was immediately centrifuged at 430 × g for 8 min at room temperature to obtain plasma that was stored at −80 °C for future analysis. For monocyte phenotyping whole blood was incubated with antibodies, namely anti-human CD14-Pac blue (Bio Legend, UK; clone M5E2), anti-human CD16-FITC (eBiosciences, UK; clone CB16) and anti-human HLADR-PE (Serotec, UK; clone: MCA1879) in the dark for 20 min at 4 °C. Post incubation red blood cells were lysed with 2 mL FACS lysing solution (Becton Dickinson, UK) and the samples incubated for 30 min in the dark at room temperature. The samples were then centrifuged at 300 × g for 10 min and washed in 2 ml phosphate buffer saline (PBS). A Cyan ADP flow cytometer was used for data acquisition and analysis. Each event was recorded in a forward‐side scatter plot in which monocytes were located according to their forward scatter profile. The gating strategy for monocyte subsets has been reported previously^54^.

### Isolation and freezing of peripheral blood mononuclear cells

Peripheral blood mononuclear cells (PBMCs) were isolated from peripheral blood by density centrifugation using Ficoll-Paque^TM^ PLUS (GE Healthcare, Sweden). Isolated PBMCs were frozen down by re-suspending cells in freezing medium consisting of 10% DMSO (Sigma Aldrich, UK) in heat inactivated fetal calf serum (Biosera, UK) and the frozen cells were then stored at −80 °C.

### Surface immunostaining of PBMCs

Frozen PBMCs were thawed at 37 °C and washed in 10 ml RPMI 1640 (Sigma-Aldrich, UK) and the pelleted cells re-suspended in PBS (1 × 10^6^ cells/ml) with any dead cells removed by centrifugation prior to immunostaining. Comparison of fresh and thawed cells from the same donor confirmed no preferential loss of specific cell types as a result of freeze-thawing (data not shown). Cells were stained with combinations of antibodies, with appropriate isotype controls used for setting gates and the percentage of positively stained cells was used to give cell frequency. The gating strategy used to identify T cell subsets^55^ and regulatory B cells, B_regs_^30^ were as reported previously.

The antibodies used were: anti-human CD3-PEcy7 (eBiosciences, clone: UCHT1); anti-human CD4 Violet (eBiosciences, clone: RPA-T4); anti-human CD8 PE (Immunotools, clone: UCHT4); anti-human CCR7 FITC (R & D systems, clone: 150503); anti-human CD45RA APC (Biolegend, clone: HI-100); anti-human CD28 APC (BD Biosciences, clone: CD28.2); anti-human CD57 FITC (eBiosciences, clone: HCD57); anti-human PTK7 (Miltenyi Biotech, clone: 188B) and anti-human CD56 PE (Milenyl Biotech, clone: AF12-7H3). For identification of B cell subsets PBMCs were stained with combinations of antibodies: anti-human CD19-PE (eBiosciences, clone: HIB19); anti-human CD27-APC (eBiosciences, clone: O323); anti-human IgD-FITC (eBiosciences, clone: IA6-2) anti-human CD24-FITC (eBiosciences, clone: eBioSN3) and anti-human CD38-PEcy7 (eBiosciences, clone: HIT2). Following incubation, cells were washed and resuspended in PBS for flow cytometric analysis using a Cyan^TM^ ADP flow cytometer (Dako).

For identification of regulatory T cells PBMCs were stained with anti-human CD3-PEcy7 (eBiosciences, UK, clone: UCHT1), anti-human CD4 Alexa fluor 450 (eBiosciences, UK, clone: RPA-T4) and anti-human CD25 APC (Biolegend, UK, clone: BC96) antibodies for 20 minutes in the dark at 4 °C. Post incubation cells were washed with PBS and re-suspended in 500 µl of Foxp3 Fix Perm Working solution (eBiosciences, UK) and incubated for 30 minutes in the dark at room temperature. Cells were then washed and resuspended in 100 µl of Foxp3 permeabilization buffer (eBiosciences, UK) and stained with anti-human Foxp3 PE antibody (eBiosciences, UK) for 30 min in the dark at room temperature. Finally, the cells were washed and resuspended in PBS for flow cytometric analysis using a Cyan^TM^ ADP (Dako Ltd, UK).

Data analysis was performed using Summit software. Spectral overlap when using more than one colour was corrected via compensation.

### Telomere length measurement

Telomere length was analysed in PBMCs using the Telomere peptide nucleic acid (PNA) kit/FITC (Dako, UK) according to the manufacturer’s instructions. Briefly, DNA from a control cell line (1301) and the test cells was denatured for 10 minutes at 82 °C in hybridisation solution with or without an FITC conjugated PNA telomere probe and then hybridised overnight at room temperature in the dark. Following two washes the sample was suspended in DNA staining solution. Sample fluorescence was measured using an Accuri C6 Flow Cytometer (BD Accuri, Biosciences) and data were analysed using C-Flow Plus Software. Relative telomere length was calculated as the ratio of the telomere fluorescence intensity of the sample and control cells, with correction for the DNA index of G0/G1 cells using the formula:

$${\rm{RTL}}=\frac{({mean}\,{FL}1\,{PBMC}\,{sample}\,{with}\,{probe}-{mean}\,{FL}1\,{PBMC}\,{sample}\,{without}\,{probe})\times {DNA}\,{index}\,{of}\,1301\,{cells}}{({mean}\,{FL}1\,1301\,{cells}\,{with}\,{probe}-{mean}\,{FL}1\,1301\,{cells}\,{without}\,{probe})\times {DNA}\,{index}\,{of}\,{PBMCs}}\times 100$$

### Statistical analysis

Statistical analysis was performed using GraphPad Prism^®^ (Graph Pad, La Jolla, USA) software. Data distribution was checked using the Kolmogorov-Smirnov test. For normally distributed data, a Student’s t test analysis was performed to assess differences between two conditions. P values of <0.05 were considered significant. A Bonferroni correction was performed to adjust for the multiple comparisons done on the three cohorts and the p values remained significant. To assess the relationship between two variable linear regression was used.

### Data availability

The primary data included in the manuscript will be made fully available upon publication.
